# Providers’ knowledge on postpartum intrauterine contraceptive device (PPIUCD) service provision in Amhara region public health facility, Ethiopia

**DOI:** 10.1371/journal.pone.0214334

**Published:** 2019-04-04

**Authors:** Yeshiwas Abebaw, Solomon Berhe, Solomon Mekonnen Abebe, Mulat Adefris, Abebaw Gebeyehu, Tadesse Gure, Birtukan Asmare, Masresha Dagnu, Wubetu Alebachew, Shumye Admasu, Solomon Abdisa, Melkamu Axumawit G/Egziabher

**Affiliations:** 1 Department of Obstetrics and Gynecology, College of Medicine and Health Science University of Gondar, Gondar, Ethiopia; 2 Department of Human Nutrition, Institute of Public Health, College of medicine and Health science, University of Gondar, Gondar, Ethiopia; 3 Amhara regional Health Biuro, Bahir-Dar, Ethiopia; 4 Department of Obstetrics and Gynecologyn, College of Medicine and Health Science Debretabor University, Debretabor, Ethiopia; Aga Khan University, KENYA

## Abstract

**Introduction:**

Postpartum intrauterine contraceptive devices (PP-IUCD) are one type of post-partum family planning method, which can be provided to a post-partum woman starting from the placental delivery time (within 10 minutes), or within the first 48 hours of postpartum period. In most developing countries, delivery time is the primary opportunity for women to access post-partum family planning methods, especially for those living in remote areas. Hence, this study assesses providers’ knowledge on postpartum intrauterine contraceptive device service provision.

**Methods:**

**A** facility-based cross-sectional study was conducted in Amhara region health center and hospitals. Health providers surveyed included obstetricians, gynecologists, general practitioners, emergency surgical officers, health officers, midwives and nurses from September 18, 2015 to December18, 2016. Simple random sampling was used to select 864 subjects. Data were collected by using a structured self-administered questionnaire and observing the facility. Multilevel analysis was done to see factors associated with outcome.

**Results:**

A total of 197 health facilities and 864 providers are included in the final analysis. Of the total providers 524 (60.6%) were from a health center. The mean age (±SD) of participants was 27.8 years (±5.4). The number of providers with good knowledge accounted for 253 of those surveyed (29.3%). The proportion of good knowledge among trained PP-IUCD providers was 35.7% (those who scored above average), and 27.9% was untrained about PP-IUCD. A considerable heterogeneity was observed between health facilities for each indicator of provider’s knowledge. Gender differences were observed as the mean knowledge score deference on PP-IUCD by 0.4 points (β = -0.41; -0.72, -0.10) when the participant was female. Having experience of regular counseling of pregnant women increases PP-IUCD knowledge score by 0.97. (**β** = 0.97; 95% CI: 0.48, 1.47). Where the health facility requested clients to purchase the IUCD themselves, the mean knowledge score decreased by 0.47 points compared with free of charge at the facility level (β = -0.47; 95%CI: -0.87, -0.07).

**Conclusion:**

Our findings showed that providers’ knowledge about postpartum IUCD was low in the Amhara region public health facility. The lowest knowledge score was noted among nurses, health Officers, midwives, and general practice professionals. Factors associated with providers’ knowledge on PP-IUCD are the status of health facility, female sex, training on PP-IUCD, regular counseling of pregnant women, and unavailability of IUCD service.

## Background

Post-partum family planning is the prevention of unintended pregnancies and closely spaced pregnancies during the first 12 months following childbirth [1]. Postpartum intrauterine devices (PP-IUCD) is an effective long-acting reversible contraceptive, which encourages women to give birth in health care facilities [2]. Compared to high-income countries, a small proportion of women receive adequate postpartum care in low income countries. Likewise, a large proportion of preventable maternal deaths occur in those countries, where the service of postpartum care is poor.

In Ethiopia, according to the emergency maternal newborn care (EmONC) report in the year 2016, about 3804 facilities were providing maternity services, and of these 876 were in the Amhara region. The number of providers who provided service for the above health facility were 26,038 (3826 general practitioners, 345 OBGYNs, 448 emergency surgical officer, 3254 midwives, 933 health officers, and 17,232 nurses) who were employed in the government and private health sector of the country. However, less than 50% of them were involved in the EmONC. In 2015, the total number of facility delivery was 1,924,330 (66%) [3]. Moreover, the government of Ethiopia aims to increase skilled birth attendance from 62 to 90% by the year 2020.

Unintended pregnancy, characterized by untimely and short pregnancy intervals can result in acute maternal complications and death of mothers and their children. Initiation of family planning at the time of birth is opportune, since few women in low-resource settings who give birth in a facility return for further care [4]. The unmet need for family planning up to a year after delivery is higher than at any other time because most women wish to delay or prevent future pregnancies at the time of the post-partum period [5]. Most of the time, delivery is the only time when mothers come in contact with the health care providers as different social, cultural and economic reasons make contact difficult (2). One study indicated that postpartum education could virtually double family planning acceptance among women who returned to the postpartum clinic within the first 9 weeks after delivery, and that the educational impact was evident irrespective of the respondents’ socio-demographic characteristics, lifestyles, and levels of readiness for family planning [6]. The low prevalence of facility delivery (66%) in Ethiopia could affect the utilization of post-partum family planning.

In 2012, an estimated 222 million women in low-resource countries wanted to avoid pregnancy, but were not using modern contraception[7]. Ethiopian Reproductive Health Strategy sets a provision for all family planning methods with special emphasis on long term & permanent methods. There are many reasons for not using contraception, specifically PPIUCD, including lack of awareness, non-availability of accessible family planning services, and limitations on women’s mobility mostly due to cultural or geographical factors[8]. Another finding has pointed out socio-demographic factors, as well as perceived attitudes towards quality of care and other organizational factors as major determinants of PPIUCD use in the country[9].

One study done in Ethiopia showed that the country has made progress in increasing its contraceptive prevalence rate from 6% to 29% between 2000 and 2011. However, the unmet need for PPIUCD remains high. In 2012, the maternal and Child Health Integrated Program (MCHIP) started a PPIUCD program implementation in Ethiopia. Currently, 15 hospitals and three health centers in Ethiopia are providing PPIUCD services. As there is a growing demand for PPIUCD, the Federal Ministry of Health recommended that with appropriate training, follow up and coaching, midwives can provide PPIUCD services effectively[10]. Effective implementation involves service strengthening that includes champions of PPIUCDs and training of providers who counsel women and attend deliveries [11].

The experience of providers when it comes to the number of IUCDs that are put in their careers shows that it advances ‘knowledge’ and ‘self-confidence’ in the capability to provide IUCD and to decrease lower age related attitudinal hindrances towards IUCD references [12]. One study shows 69–78% of providers have good factual knowledge about the IUCD and felt adequately prepared to insert a device or counsel women about it [13]. They recommended the provision of training on PPIUCD in order to increase knowledge and skills among health care providers to further promote PPIUCD use and aid in reduction of the expulsion rate [14]. Though it is the best strategy to alleviate the problem of unwanted pregnancies, use of postpartum contraception is low in developing countries including Ethiopia. Long acting contraceptive methods like IUCD are believed to be effective methods during the postpartum period. There are client and provider side factors contributing to the limited use of these methods. One of the major provider side factors for low/limited PPIUCD contraceptive use is providers’ experience and knowledge. Provider knowledge has not been previously assessed in this study area, moreover, understanding deficiencies in providers’ knowledge and experience could be an actionable intervention strategy for possible interventions like in-service training and provision of supplies. Therefore, this study will identify sociodemographic, medical training profile, length of time working in a medical facility, and work-related site levels of knowledge among health providers to try to identify the knowledge gap and recommend interventions based on the findings.

## Methodology

### Study design and period

A Facility-based cross-sectional study was conducted at Amhara region public health facility from September 18, 2015 to December18, 2016.

### Study area and subjects

The Amhara region is located in the northwestern and north central part of Ethiopia. The capital city is Bahir-Dar. In 2014, the region had an estimated total population of 19,602,512. Among this, 23.58% represent women aged 15–49 years, and pregnant women constitute about 3.24%. About 86.1% of the population is estimated to be rural inhabitants. The region has 19 hospitals, 801 HC HC) & 3302 health posts. All health workers who were providing maternity service in health facilities in the Amhara region (hospitals and health centers) were source population. Whereas, the study population were all health workers who were providing maternity services in selected health facilities during the study period. This study involves at list BSc degree holder those include registered Nurses, midwives and Health officers (health practitioners like a medical doctor they provide clinical service at a primary health care and public health service at a community), and General practitioners (registered Medical doctor but not specialized).

### Sample size and sampling procedure

The sample size was determined using a single population proportion formula. The assumption includes; 5% margin of error, 95% confidence interval and 50% of the providers represent proportion (p) with good knowledge on PPIUCD. Considering a design effect of 2 and a non-response rate of 10%, the total sample size was 864 health care providers (level 2). The facilities were selected randomly and all the eligible providers were included for the study. From the total, 19 hospitals and 520 health centers available in Amhara region, 10 hospitals and 149 health centers were selected using simple random sampling (lottery method) after obtaining the latest list from the region health administration (level 1). Secondly, eligible health personnel were represented proportionally within each group (from hospitals 12 to 16 and health centers 2 to 6 providers). Finally, the selected eligible adults in each health center were sampled using simple random sampling.

### Data collection

A structured knowledge questionnaire was developed to assess knowledge, and its reliability was established. A self-administered pretested and structured questionnaire was used as the instrument of data collection from providers perspective. This questionnaire included questions on socio-demographics, service delivery, family planning counseling and knowledge assessment. Similarly, individual facility observation was done using a pre-structured checklist for facility auditing. The selection of data collectors was made from health practitioners outside the working facility. Training was conducted for 30 data collectors (degree level health officers, midwives and nurses) and 9 supervisors, regarding the objectives of the study and how to collect data. Additionally, two days of pre-testing were set up.

### Variables of the study

The outcome variable for this study was providers’ knowledge. We computed knowledge from the 12-knowledge score question and we use the outcome as a continuous variable for the model after checking the linear regression assumption. For the descriptive report, after computing all the 12 variables, we also categorized knowledge in two categories (participants whose knowledge mean score was at or above the mean 50%, were considered as good knowledge and the one who score below 50% are considered as having poor knowledge). The independent variables in this study were: profession/qualification of provider (obstetrician and gynecologist, OBGYN residents, emergency surgical officers (ESO), general practitioners, midwives, health officers and nurses) and socio-demographic variables (sex, age, religion). We also gathered information on facility related variables including availability of FP service delivery guidelines/clinical management protocols, examination room or area providing client privacy room for screening, counseling, and examination, examination table, storage area or cupboard for drugs and other supplies, speculums (various sizes), stretchers, special IUCD inserter clamp, and sponge forceps, to see the attributable effect on knowledge at the facility level.

### Data analysis procedure

Data were coded, entered and cleaned, using EPI-INFO version 7 and STATA version 14 statistical package. Frequencies, percentages, cross-tabulations and Chi-square tests for categorical variable and mean, standard deviation, and range for a continuous variable were used. Descriptive statistics were used to summarize the data. Multilevel modeling was applied to identify facility and provider level determinants. To determine the relationships between provider level and facility level factors associated with providers knowledge, we used a multilevel linear regression. Our assumption was providers knowledge can be influenced by facility level quality of service. We ran three models: an empty model (null model) without covariates; a model containing only facility level variables; and a model comprising both the provider and facility level variables to see if the quality of care in PPIUCD services is influenced by the knowledge of health care providers at different health facilities levels. This will help to consider the individual provider level and the facility-level in the same analysis, to see correlation between providers within the same facility. The collected data had a hierarchical structure where providers’ data nested in the facility.

Linear regression was applied to determine factors associated with knowledge. Intra-class correlation in the empty model was assessed for heterogeneity after accounting for facility level variables, and random effects was also used to test the variation within the provider and across all facilities.

### Ethical considerations

Ethical clearance was obtained from the Institutional Review Board (IRB) of the University of Gondar before starting the actual data collection. Permission was obtained from Amhara Regional Health Bureau, Zonal Health Departments/district health offices and health facility managers. The study participants were informed about the purpose of the research. Signed written consent was obtained from all study participants. The privacy and confidentiality of the participants were not disclosed, and their right to withdraw at any time from the study was informed and respected.

## Result

A total of 197 health facility and 864 providers are included in the final analysis. Of the total providers, 524 (60.6%) were from health centers and had a mean age (±SD) of 27.8 years (±5.4). Of these, female participants accounted for 337 (39%) of all providers. The proportion of females in the health center was 44.3% and 30.9% at the hospitals. About 61.7% of the study participants had less than 5 years’ work experience as health profession, with a range of 1 to 40 years. The socio-demographic characteristics of the study participants are presented in Table 1.

**Table 1:** Socio-demographic characteristics of the study population by health facility in Amhara regional state, Ethiopia, 2016.

Variable	Frequency	Percentage
**Type of Facility**		
Hospital	524	60.6
Health center	340	39.4
**Providers sex**		
Male	527	61
Female	337	39
**Providers age (Years)**		
18 to 24	207	23.9
25 to 34	570	65.9
35 to 44	65	7.5
45 to 69	22	2.6
**Marital status**		
Currently Married	394	45.6
Not Married	470	54.4
**Religion**		
Orthodox	705	81.6
Muslim	117	13.5
Protestant	21	2.43
Other	21	2.43
**Profession**		
Gynecologist	5	0.58
Residence	10	1.16
G. practitioner	65	7.52
Emergency surgery	48	5.56
Health officer	105	12.15
Midwife	341	39.47
Nurse	290	33.56
**Duration of Experience**		
Less than 5years	481	61.7
5 to 40 years	299	38.3

**Fig 1:**
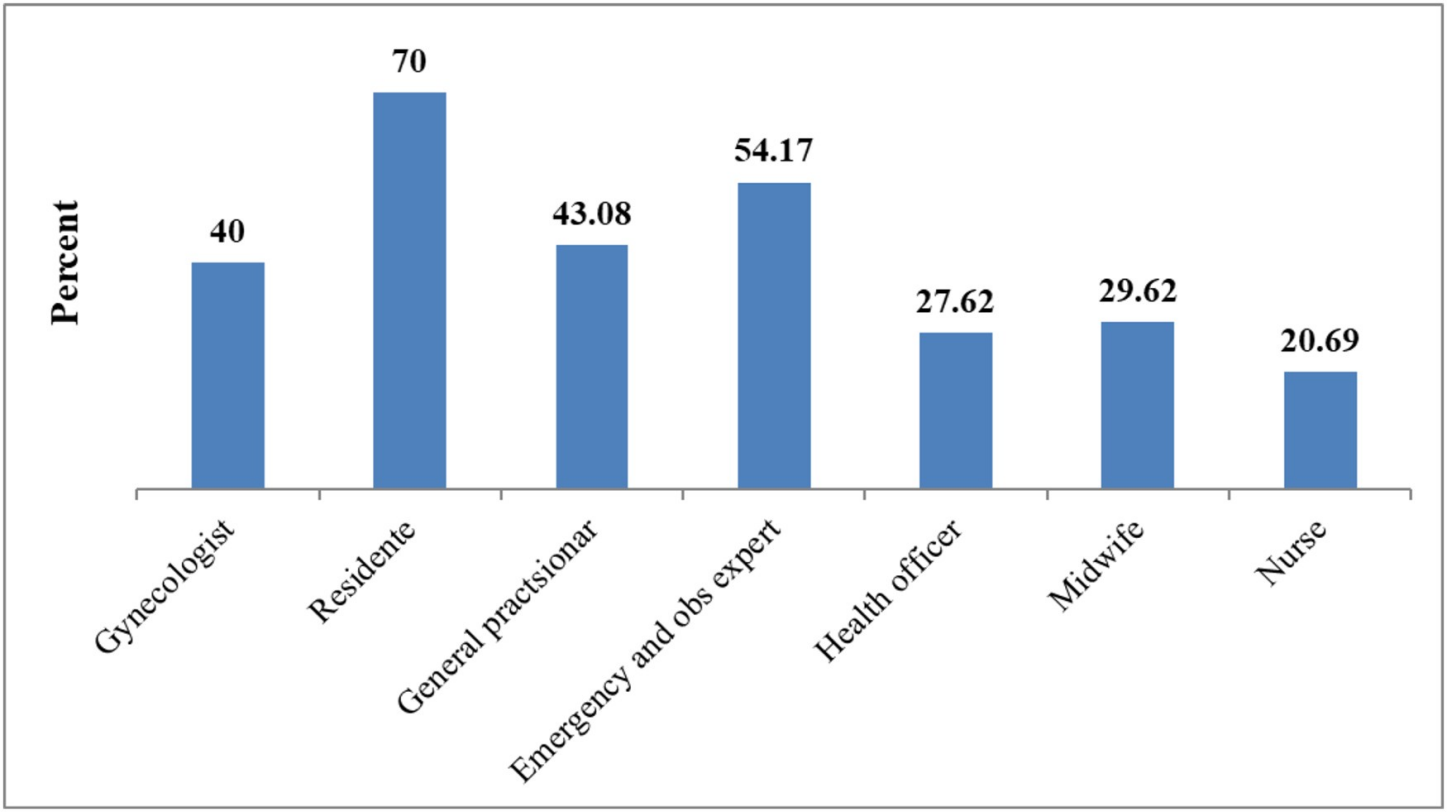
The proportion of good knowledge (50% and above) by profession in PPIUCD in Amhara regional state health facility, Ethiopia, (2016).

**Fig 2:**
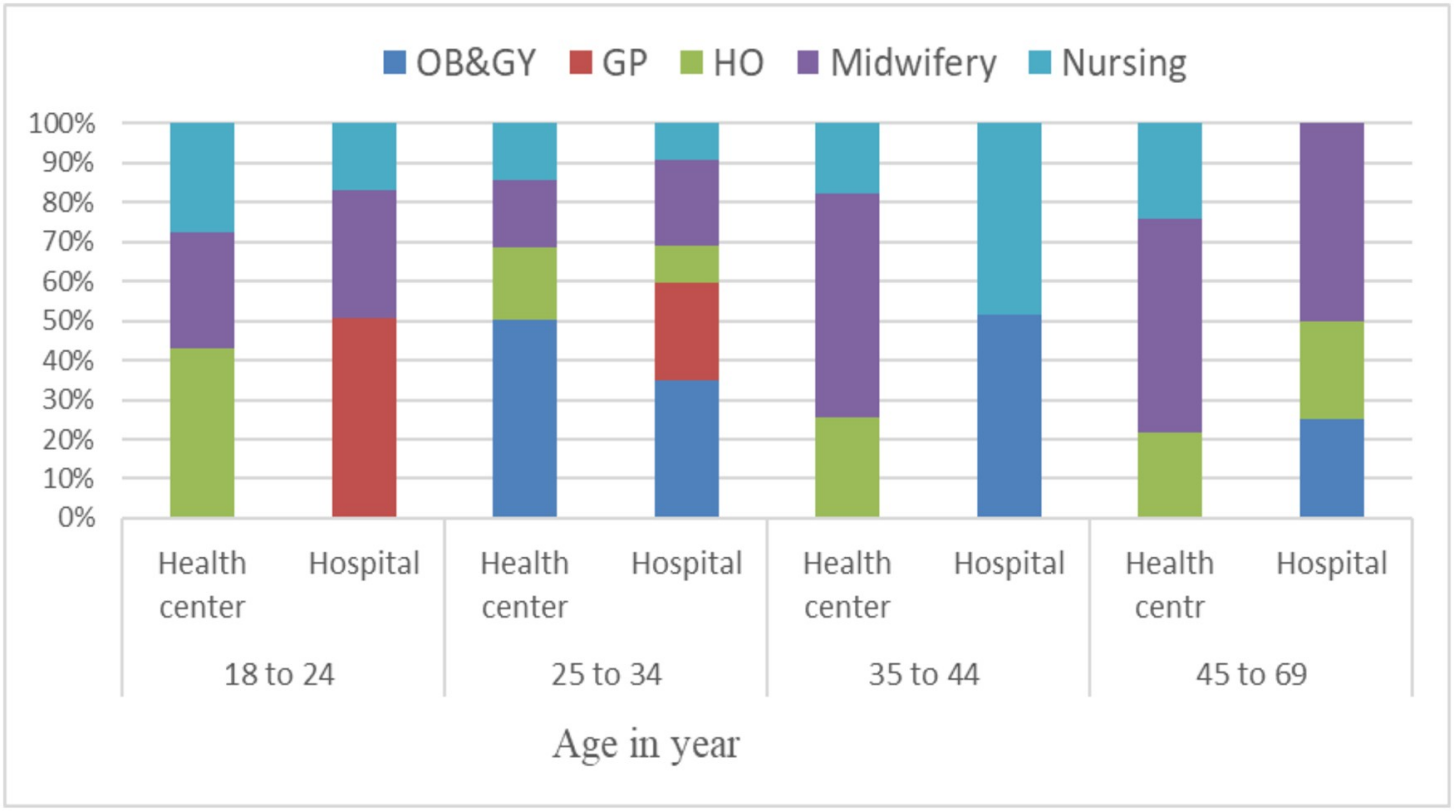
The proportion of good knowledge by profession and Health facility in PPIUCD in Amhara regional state health facility, Ethiopia, (2016).

The percentage mean (±SD) knowledge score was 34.98 (±SD 19.4) with a range between 0 to 91.67. The proportion of good knowledge (individuals who scored 50% and above) accounted for 253 (29.3%). The proportion was high among providers who were trained PP-IUCD, 35.7% than that of untrained (27.9%) (Table 2). In terms of profession, 70% of OBGYN residents had good knowledge about PP-IUCD. The lowest knowledge score was noted among nurses (20.7%) (Fig 1). The Percentage of good knowledge was higher among males (33.2%) than women (23.1%). The proportion of knowledge was higher in hospital providers 36.76% (95%CI: 31.79, 42.04) compared with health center providers 24.42% (95%CI: 20.93, 28.29) (Fig 2).

**Table 2:** Provider knowledge and practice in family planning service among Amhara regional state health facility, Ethiopia, (2016).

Variable	Frequency	Percentage
**IUCD insertion skill**		
No	21	14.3
Yes	126	85.7
**Family counselling**		
No	18	12.7
Yes	124	87.3
**ANC**		
No	4	2.04
Yes	192	97.9
**Normal delivery care**		
No	3	1.52
Yes	194	98.5
**Early post-partum check-up**		
No	10	5.1
Yes	186	94.9
**Normal delivery care**		
No	11	5.58
Yes	186	94.4
**Advise support & promotion**		
No	4	2.03
Yes	193	97.97
**Counselling maternal self-care**		
No	4	2.04
Yes	192	97.96
**Skilled attendant for maternal care**		
No	13	6.6
Yes	184	93.4
**Caesarean section service**		
No	133	67.5
Yes	64	32.5

The proportion of nurses, health officers, midwives and general practitioners was 801(92.7%) off this only 149 (17.2%) of them had received PP-IUCD training.

Regarding counseling, 90.2% of the health providers reported to regularly counsel pregnant mothers on family planning, of these 75.5% of them counsel at antenatal care, 29.9% of them counsel during labor, 58.5% of them counsel immediately after delivery, and 67% of them counsel during postnatal visits.

About 78% of the health providers regularly counsel about IUCD, 4% of them counsel during labor, 19.9% counsel right after delivery, and 20.5% counsel during postnatal visit.

About 59.8% of staff from the visited health facilities were trained in interval IUCD insertion skills within the past year. About 66.5% of health facilities’ flow charts were easily available for staff, in 4% of the health facilities the flow charts are locked and are difficult to get, and in 7% of the health facilities the flow charts are kept at home.

From the observations, 60.9% of the facilities family planning room is observed to be available and satisfactory, and in 6.1% it is not available. In 78.7% of the health facilities delivery bed/couch is observed to be available and satisfactory and in 2.5% the health facilities it is observed to be unavailable.

In 56.4% of the health facilities IEC teaching and counseling materials on post-partum care are seen. About 77.2% of the interviewed health professionals said that they regularly counsel pregnant or postpartum women on family planning (Table 3).

**Table 3:** Health facility characteristics of the study area in Amhara regional state, Ethiopia, 2016.

Variable	Frequency	Percentage
**Interviewed Facility Position**		
Medical director	15	7.61
Head of Health facility	150	76.1
Other profession	32	16.24
**Facility Head qualification**		
Specialist	1	0.51
Health officer	54	27.4
GP	17	8.63
Nurse	87	44.2
Other	38	19.29
**People from outside this facility seek care**		
Never	4	2.03
Rarely	55	27.9
Frequently	128	64.9
Do not know	10	5.08
**Professionals in the facility**		
Physician	644	6.95
Health officer	484	5.22
Nurse	3742	40.4
Midwife	911	9.83
Anesthetist	1688	18.2
Laboratory	98	1.06
Pharmacist	783	8.45
Environmental health	327	3.53
Other(non-health)	587	6.34

### Factors associated with PP-IUCD knowledge

The multilevel analysis started from the intercept-only model to test the null hypothesis that there was no variation in knowledge among health facilities, and to evaluate the random effects at the health facility level. The results indicated that a considerable heterogeneity was observed among health facilities for each indicator of provider’s knowledge. The intra-class correlation in the empty model for knowledge by health service provider indicated that 20% of the total variance in knowledge was attributable to the differences across health facilities (Table 4).

**Table 4:** An empty model for multilevel analysis for facility level factors associated with PP-IUCD Knowledge in Amhara regional state health providers Ethiopia (2016).

Knowledge	Coef.	Std. Err.	z	P-value	[95% CI:]
_cons	4.141	0.106	39.08	< 0.001	[3.93, 4.35]
**Random-effects Parameters**	Estimate	Std. Err			
**Health facility Identity**					
Var (cons)	**1.098**	0.223			[0.74, 1.64]
Var (Residual)	**4.342**	0.235			[3.90, 4.83]

Female sex decreases the mean knowledge score on PP-IUCD by 0.4 points (β = -0.41; -0.72, -0.10); The mean score of PP-IUCD knowledge were reduced by 0.9 in general practitioners, 1.3 in health officer, 1 in midwives, and 0.5 in nurses, compared with other health workers (obstetrician and gynecologists, and ESO), (β = -0.88; 95%CI: -1.67, -0.83), (β = -1.32; 95%CI: -2.07, -0.57), (β = -1.00; 95%CI: -1.65, -0.35), (β = -0.50; 95%CI: -2.16, -0.83), respectively. Having experience of regular counseling of pregnant women increased the PP-IUCD knowledge score by 0.97. (**β** = 0.97; 95% CI: 0.48, 1.47). Health facilities requesting that clients purchase the IUCD during the service decreased the mean knowledge score on PP-IUCD by 0.47 points compared with free of charge (β = -0.47; 95%CI: -0.87, -0.07) (Table 5).

**Table 5:** Multivariate analysis for factors associated with PP-IUCD Knowledge in Amhara regional state health providers Ethiopia (2016).

Variable	Good Knowledge	Crud Beta coefficients [95%:CI]	Adjusted Beta coefficients [95%:CI]
**Variables at Providers level**			
**Age in year**			
18 to 24	52 (25.1)	1.00	
25 to 34	174 (30.5)	0.14 [-0.22, 0.49]	0.07 [-0.29,0.43]
35 and above	27 (31.03)	0.17 [-0.39, 0.74]	0.162 [-0.48, 0.71]
**what is your sex**			
Male	175 (33.2)	1.00	1.00
Female	78 (23.2)	-0.54 [-0.84, -0.23]*	-0.41 [-0.72, -0.10]*
**Current Profession**			
Gynecologist, Resident and Emergency surgical officer	35 (55.6)	1.00	1.00
GP	28 (43.1)	-0.80 [-0.59, -0.022]*	-0.88 [-1.67, -0.83]*
HO	29 (27.6)	-1.56 [-2.28, -0.84]*	-1.32 [-2.07, -0.57]*
Midwifery	101 (29.6)	-1.09 [-1.70, -0.49] *	-1.00 [-1.65, -0.35]*
Nursing	60 (20.7)	-1.71 [-2.35, -1.09]*	-0.50 [-2.16, -0.83]*
**Have you been trained in PPIUCD ins**			
No	197 (27.9)	1.00	1.00
Yes	56 (35.7)	0.67 [0.28, 1.06]*	0.36 [-0.10, 0.82]
**Do you regularly counsel pregnant**			
No	16 (19.1)	1.00	1.00
Yes	234 (30.3)	1.09 [0.60, 1.58*]	0.97 [0.48, 1.47]*
**Have you ever insert PP-IUCD**			
No	213 (27.9)	1.00	1.00
Yes	40 (40.0)	0.93 [0.46, 1.39]*	0.53 [-0.01, 1.07]
**Did talk about IUCD during PNFP**			
No	78 (25.2)	1.00	1.00
Yes	175 (31.6)	0.31 [-0.004, 0.63]	0.16 [-0.15, 0.48]
**Variables at a Facility Level**			
**Are there Service for Caesarean section every day in the facility**			
No	114 (24.5)	1.00	1.00
Yes	139 (34.8)	0.53 [0.12, 0.95]*	0.18 [-0.26, 0.62]
**Are FP clients required to purchase /provide the IUCD**			
No	183 (31.1)	1.00	1.00
Yes	66 (25.2)	-0.59 [-1.02, -0.15]*	-0.47 [-0.87, -0.07]*
**Type of health**			
Health center	128 (24.4)	1.00	1.00
Hospital	125 (36.8)	0.59 [0.17, 1.01]*	0.22 [-0.26, 0.69]

* = P-value < 0.05

## Discussion

Across the world, particularly in developing countries, the use of long acting reversible forms of contraceptive methods, especially postpartum intrauterine device (PPIUCD) is being promoted largely in the postpartum period (are commonly used to refer to the first 6 weeks following childbirth). This study had several purposes. First, we tried to establish a baseline of health service provider’s knowledge, regarding PPIUCD in the Amhara region of Ethiopia. Secondly, we evaluated the availability of health service delivery facilities and their preparedness for the PPIUCD service delivery in the same region. In this study, the proportion of knowledge about PPIUCD was generally poor compared with other developing countries having similar health facilities. A study done in North Carolina, one of the fastest growing Mexican-American populations, showed knowledge on postpartum IUCD among health care providers to be minimal on how to address the family planning needs of this population [15]. A major gap in knowledge concerns demand for, and means of promoting, immediate postpartum family planning services in Asia and Africa. Counseling before discharge is likely to have an impact on subsequent contraceptive uptake [16].

In this study the lowest knowledge score was noted among nurses, health officers, midwives, and general practitioners. Knowledge about PPIUCD had higher quality indicators for providing information on PPIUCD. However, in our findings, knowledge about PPIUCD was found to be poor among nurses, health officers, midwives, and general practitioners, professionals who are the frontline health workers in routine family planning service. A study also revealed that a sizable amount of postpartum pregnancies are unplanned which is closely correlated with no prior knowledge to of use of such medication and a fear of contraceptive use in low-income settings. [17]. Increasing the level of awareness was an important significant predictor of unmet need. A study done India showed a majority (69%-78%) of providers had good factual knowledge and felt adequately prepared to insert a device or counsel women about it [18].

There were knowledge gaps that were related to PPIUCD counseling and in other findings these gaps were closely related to the use of PPIUCD amenities. In spite of the fact that women were more likely to be given information about the risks and alternatives to PPIUCD in the health facility, which intended to increase the likelihood of acceptance of PPIUCD insertion [19]. Therefore, the study findings call for efforts that improve the training of midwives, nurses, health officers and general practitioners who provide PPIUCD counseling at the family planning clinics and delivery services. Finings revealed that a postpartum educational effort could almost double family planning acceptance among women who returned to the postpartum clinic within the first 9 weeks after delivery. Additionally, the educational impact was evident irrespective of the respondents’ socio-demographic characteristics, lifestyles, and levels of readiness for family planning [6].

Despite the well-established benefits of PPIUCD, access to PPIUCD can be affected by affordability and availability of the PPIUD. In this study we were able to uncover that in some of the visited health facilities, the clients are forced to purchase an IUCD, this may limit IUCD use, as its uptake continues to be low in the study area. The Amhara region, which is the primary area of the study has an observed demand and supply imbalance when it comes to primary health facility’s supplies. One of the reasons could be the insufficient budget which effects medical supplies in the public health system that may directly affect the availability of IUCDs and other medical supplies. The Ethiopian Government has specified that family planning services, including IUCDs, are free of charge however during our observation the supplies were not sufficiently available. The subsidizes were not able to keep up with the constant demand hence the supply’s underutilization in the area is directly impacting the IUCD use. When this is the case the individuals will be forced to purchase these IUCDs from private dealers which is a great strain for them due to their financial difficulties. The Ethiopian government needs to allocate an increase in budget to the public health sectors to ultimately minimize high fertility rates and maternal mortality rates in the countries. Studies done in Sri Lanka, Tanzania, and Nepal showed PPIUCD offers a convenient and cost-effective postpartum contraceptive option for women who do not get the service because of cost, distance, or health system challenges [20]. Researchers in Sub-Saharan Africa have found that health facility factors influence client contraceptive use [21].

In the multilevel analysis model, the results indicated that considerable heterogeneity was observed among health facilities for each indicator of provider’s knowledge. That is, the total variance in knowledge was attributable to differences across health facilities. This is not in line with WHO task-sharing guidelines and the desire for more evidence on the capabilities of lower level health workers [21]. Providers who get frequent training in PPIUCD in the immediate postpartum period will increase the likely hood of IUCD practice, which is supported by a report from Ghana [22]. This finding implies that lots of work should be done and more emphasis should be given on providing training and support to the health service providers of different levels from different health facilities to improve their knowledge, manage side effects, and recognize women in periods of high unmet need-such as post-partum or post-abortion women-as suitable candidates for IUCDs [23].

In this study, female sex showed a relatively lower mean knowledge score on PPIUCD as compared to the male practitioners. This finding may be explained by the profession differences among the providers, as most of the females involved in the study were midwives and nurses who were working in health centers mostly in the country sides where their involvement in the teaching or training activities is less likely compared to health professionals from higher health institutions where most of them are found to be males in our study. This is in contrast to findings from other study, which showed provider’s knowledge was not correlated with the provider’s position as a nurse or midwive [24] and as was discussed previously the active role of health care providers in teaching and training forums have a sizable impact in increasing the knowledge score of the providers. And in the Nepalese scholarly work, IUCD knowledge was crucially related with providers previous training[25]. Therefore, providing training could help to improve their knowledge about PPIUCD.

There are different factors that will determine the uptake of the PPIUCD service. Among these factors, this study identified having experience with regular counseling of pregnant women increases PPIUCD knowledge score. This is in line with the study from South Africa that showed the positive effect of skills training impact on the overall performance of the providers, including their attitude on counseling and their overall knowledge and skills. [24]

Health facilities requesting clients to purchase an IUCD to provide PPIUCD during the service decreases the mean knowledge score on PPIUCD compared with free of charge at the facility level data. This study showed non-availability of the IUCD in the facility for free is shown to decrease the uptake of the service delivery. This will have repercussions for the service and the practitioners, as the practitioners in these sites will not be kept motivated and active towards improving their knowledge and practice. Contrary to this a study, in Addis Ababa, Ethiopia, a study indicated that availability of the IUCD was not a problem in any of the 22 health facilities (8, hospitals and 12 HC) [26]. Routine quality control and supportive visits can be used to further improve the knowledge and attitude among providers.

## Conclusion

In our findings, providers’ knowledge about postpartum IUCD was low in the Amhara region public health facility. The lowest knowledge score was noted among nurses, Health Officer, midwives, and general practice professional. Overall, counseling services for PPIUCD right after delivery was low. About half of staff from the visited health facilities were trained in interval IUCD insertion skills within the past year, and the majority were providing postpartum care service on a daily basis. Of the visited health facilities, clients’ need to purchase an IUCD had an effect on IUCD utilization.

## Recommendation

From this study, we understand that there is a major knowledge gap among the health service providers, and for this the ministry of health of Ethiopia has already developed and issued a comprehensive PPIUCD training manual for training of health professionals in 2013. However, practical implementation of the training at the different regions of the country should get more emphasis and more work is expected for its implementation. Providers from remote areas should get well trained and get updated with possible on-site trainings for successful delivery of the PPIUCD service. FP interventions need target nurses, health officers, midwives and general practitioners in order to have great impact by the right skills and reaching more people. For the successful delivery of the PPIUCD service, the health facilities in Ethiopia should be well equipped with every necessary material, for this, stakeholders at every level should be responsible to make the necessary materials available in the respective health facilities.

## Supporting information

S1 File Study tool. (DOCX)
