# Supplementary material for: Providers’ knowledge on postpartum intrauterine contraceptive device (PPIUCD) service provision in Amhara region public health facility, Ethiopia
Source: PLoS One. 2019 Apr 4;14(4):e0214334. doi: 10.1371/journal.pone.0214334 (PMC6449033; doi:10.1371/journal.pone.0214334)
Supplement: S1 File — (DOCX) [file pone.0214334.s001.docx]

Study tool (support document)

#

|  | District | Facility | | Facility head | Data collector |
| --- | --- | --- | --- | --- | --- |
| Name |  |  | |  |  |
| ID (code) |  |  | |  |  |
| Date of data collection_____________ | | | Time: from ______to_________ | | |

# Annex 2: Questionnaire

| **Provider identification code:__________________** | | | | | |
| --- | --- | --- | --- | --- | --- |
| **I. Background information** | | | | | |
| **Ser.No** | **Questions** | **Response** | | | **Code** |
| 101. | Age of the respondent | ________ | | | A1 |
| 102 | What is your Sex? | 1. Male  2. Female | | | A2 |
| 103 | What is your Religion? | 1. Orthodox 2. Muslim 3. Protestant 4. Catholic 5. Others specify………….. | | | A3 |
| 104 | What is your profession? | 1.Gynecologst/Obstetrician  2. General practitioners  3. IEOS  4. Health Officer  5. Midwife  6.Nurse | | | A4 |
| 105 | How is your Marital status? | 1.maried  2. Not married  3. divorced  4. widowed | | | A5 |
|  | | | | |  |
| **S. No.** | **Questions** | **Responses Code** | | |  |
| 106 | In what year did you complete your basic training? Year of graduation | _________________ | | | A6 |
| 107 | Were you trained in postpartum IUCD (PPIUCD) insertion? | 1. Yes 2. No | | | A7 |
|  | 107.a by whom? | Trained by_______ | | | A7a |
| 108 | If Yes to Q. no 107, when? How many months or years back? | ___________  month/year back | | | A9 |
| II. Service Delivery | | | | |  |
| 201 | Have you ever inserted an IUCD? | 1.Yes  2.No | | | B1 |
| 202 | If Yes to the above question, how many IUCDs did you insert in the past 30 days? | _______________ | | | B1a |
| 203 | Of those how many were | 1.post placental within 10  min after delivery______  2. intracecarean_______  2. immediate post delivery  within 48 hours of delivery________  3.extended postpartum 4  Wks post delivery______ | | | B1b |
|  | If No to question no201, what is your main reason not to insert | 1. I have no Training 2. I have no experience 3. Lack of necessary instrument 4. Client refusal 5. Other specify…... | | | B1c |
| 204 | Have you ever inserted a PPIUCD? | 1. Yes 2. No | | | B2 |
|  | If no to the above question what is your main reason not to insert? Specify shortly | 1. I have no training 2. Ihave no experience 3. The appropriate material are not available 4. I feel it is not safe as compared to other methods 5. Patients don’t accept it | | | B2a |
| 205 | In your experience, when is the ideal time for a postpartum woman to have an IUCD inserted?( MULTIPLE ANSWER IS POSSIBLE) | 1. Post-placental 2. Immediate postpartum 3. Extended post partum 4. It doesn’t matter 5. Other, specify | | | B3 |
| 206 | When you perform post-placental IUCD insertion, do you prefer manual or instrumental insertion? | 1.manual  2.instrumental  3.no preference | | | B4 |
| III. Family Planning Counseling | | | | | |
| 301 | In your opinion, what FP methods are most suitable for postpartum women?( MULTIPLE ANSWERES ARE POSSIBLE) | 1. Female sterilization 2. Male sterilization 3. Pill specify( POP or COC) 4. IUCD 5. Injectables 6. Implants 7. Male condom 8. Female condom 9. Diaphragm 10. LAM | | C1 | |
| 302 | Do you regularly counsel pregnant or postpartum women on FP? | 1.YES  2.NO | | C2 | |
| 303 | When do you counsel pregnant or postpartum women on FP?( MULTIPLE ANSWERES ARE POSSIBLE) | 1. at ANC  2. in labor  3.Right after Delivery  4. During PNC visits  5. Other specify | | C3 | |
| 304 | Do you regularly talk about IUCD when you counsel pregnant or post partum women on FP? | 1.Yes  2.No | | C4 | |
| 305 | In your experience, when is the best time to counsel pregnant or postpartum women on PPIUCD? | 1.at ANC  2. In Labor  3. Right after delivery  4. During post natal visit  5. Other, Specify… | | C5 | |
| 306 | In your experience when do pregnant or postpartum women make their decision about FP in general? | 1.at ANC  2. In Labor  3. Right after delivery  4. During post natal visit  5. Other, Specify… | | C6 | |
| IV. Knowledge assessment | | | |  | |
| 401 | The unmet need for family planning among postpartum women during the first year postpartum is? | 1.Higher than the unmet  need for other women  2.Same as the unmet  need of other women  3.Lower than the unmet  need for other women | | D1 | |
| 402 | Which of the following is TRUE about the expulsion of the postpartum IUCD? | 1.Choosing the correct  type of IUCD will  reduce expulsion rates?  2.Proper insertion  technique reduces  the expulsion rate.  3.Expulsion rates are  lower in low party women. | | D2 | |
| 403 | Which of the following is NOT an acceptable time to insert an IUCD immediate postpartum? | 1.20 minutes after  expulsion of placenta  2. 36 hours postpartum  3. 2 weeks postpartum | D3 | | |
| 404 | Because of the normal postpartum changes | 1.The string should be  trimmed before insertion  of the IUCD  2.The women should  check for expulsion of  the IUCD after breastfeeding  3.The woman is less  likely to notice initial  slight bleeding and  cramping caused by  the IUCD | D4 | | |
| 405 | Which of the following is TRUE about counseling a woman for immediate postpartum IUCD? | 1.Perforation rate are  very high with  immediate postpartum  IUCD insertion  2.It is not a good  method for postpartum  woman.  3.Many women may report  missing strings during first  follow-up visit. | D5 | | |
| 406 | When should a clinician start counseling a woman for immediate postplacental IUCD insertion? | 1.During the antenatal  period, so she can make  a clear decision  2.Before pregnancy,  so the she has the  ability to consider her  reproductive plans  3.Only if the women  requests counseling, to  ensure she has a free choice | D6 | | |
| 407 | For which of the following clients is the immediate postpartum IUCD NOT a good contraceptive choice? | 1.woman with HIV infection  who is on antiretroviral  therapy  2.A woman who was  treated for purulent  cervical discharge in the  third trimester  3.A past history of  ectopic pregnancy | D7 | | |
| 408 | Which of the following is the best technique to insert an IUCD within 48 hours of childbirth? | 1.Using a Kelly placental  forceps or ring forceps  2.Manually  3. Using an inserter tube and plunger | D8 | | |
| 409 | In order to ensure that the IUCD is placed at the fundus and stays there, the clinician inserting the IUCD should | 1.Perform manual insertion  , so that he/she can feel  the IUCD is at the fundus.  2.Release the IUCD  from the forceps at the fundus  and move the forceps to  the side before withdrawing  the forceps  3.Apply countertraction by  holding firmly on the anterior lip of the cervix while moving the IUCD directly upward toward the fundus | D9 | | |
| 410 | The technique of inserting an IUCD during a cesarean section includes all of the following EXCEPT? | 1.Place the IUCD high  in the fundus of the uterus,  either using a hand  or an instrument  2.Pas the strings of the  IUCD through the cervical  canal  3.Close the uterine  incision carefully to ensure  that the strings are not  entangled in the uterine repair. | D10 | | |
| 411 | A woman who has had an IUCD placed in the immediate postpartum period should have a follow-up exam | 1.After six weeks to  reinforce counseling and  answer any questions  2.Every year to check  for the strings  2.Only if she feels the IUCD  has fallen out | D11 | | |
| 412 | During every follow up visit the providers should do the following with the woman: | 1.Tell the woman for how  long she should keep her  IUCD  2.Reviewher understanding  of the IUCD and ask if she  has any questions.  3.Perform a pelvic exam to  look for the strings or a  partially protruded IUCD. | D12 | | |

##

## **TOOLS FOR THE FACILITY BASED SURVEY**

### Facility based survey questionnaire

|  | District | Facility | | Facility head | Data collector |
| --- | --- | --- | --- | --- | --- |
| Name |  |  | |  |  |
| ID (code) |  |  | |  |  |
| Date of data collection_____________ | | | Time: from ______to_________ | | |

| **Facility manager identification code:___________** | | | | | | | | | | | | | | | | | | | | |
| --- | --- | --- | --- | --- | --- | --- | --- | --- | --- | --- | --- | --- | --- | --- | --- | --- | --- | --- | --- | --- |
| **PART I Interview of facility manager (officer in charge of the facility)** | | | | | | | | | | | | | | | | | | | **Code** | |
| 101 | **What is your position?** | | | | | | 1. Medical director 2. Head of the facility   Other (specify): _________ | | | | | | | | | | | | E1 | |
| 102 | **What is your qualification?** | | | | | | 1. Specialist 2. Health Officer 3. General practitioner 4. Nurse   Other (specify): _________ | | | | | | | | | | | | E2 | |
| 103 | **What is the estimated catchment population served by this facility?** | | | | | | Total: _________  99 Do not know | | | | | | | | | | | | E3 | |
| 104 | **In your estimation, how frequently do people from outside this facility catchment area seek care here?** | | | | | | 1. Never 2. Rarely 3. Frequently   99.Do not know | | | | | | | | | | | | E4 | |
| 105 | **How many beds are there at this facility (including maternity)?** | | | | | | Total: _________ | | | | | | | | | | | | E5 | |
| 106 | **How many maternity beds are there at this facility?** | | | | | | Total: _________ | | | | | | | | | | | | E6 | |
| 107 | **What is the antenatal care coverage rate for this facility in the previous year?** | | | | | | _________ %  99. Do not know | | | | | | | | | | | | E7 | |
| 108 | **What is the delivery coverage rate for this facility in the previous year?** | | | | | | _________ %  99. Do not know | | | | | | | | | | | | E8 | |
| 109 | **What is the postpartum care coverage rate for this facility in the previous year?** | | | | | | _________ %  99. Do not know | | | | | | | | | | | | E9 | |
| **STAFFING** | | | | | | | | | | | | | | | | | | |  | |
| **At this facility how many of each of the following staff are employed and working?** | | | | | | *Number of posts occupied:* | | | | | | | | | | | | |  | |
| 110 | **Physicians (both general medicine and obstetrician/gynecologist)** | | | | | Total: _________ | | | | | | | | | | | | | F1 | |
| 111 | **Health officers** | | | | | Total: _________ | | | | | | | | | | | | | F2 | |
| 112 | **Nurses** | | | | | Total: _________ | | | | | | | | | | | | | F3 | |
| 113 | **Midwifes** | | | | | Total: _________ | | | | | | | | | | | | | F4 | |
| 114 | **Health extension workers** | | | | | Total: _________ | | | | | | | | | | | | | F5 | |
| 115 | **Anesthetists** | | | | | Total: _________ | | | | | | | | | | | | | F6 | |
| 116 | **Laboratory staff** | | | | | Total: _________ | | | | | | | | | | | | | F7 | |
| 117 | **Pharmacists** | | | | | Total: _________ | | | | | | | | | | | | | F8 | |
| 118 | **Sanitarians(ENV.H workers)** | | | | | Total: _________ | | | | | | | | | | | | | F9 | |
| 119 | **Other (non-health worker staff—guards, cleaners, etc.)** | | | | | Total: _________ | | | | | | | | | | | | | F10 | |
| **RECENT TRAINING** | | | | | | | | | | | | | | | | | | | **G** | |
| 120 | | | **How many staffs at this facility have received training in** Family Planning **(in-service/continuing education) within the past twelve months?** | | | Total: _________  99. Do not know | | | | | | | | | | | | | G1 | |
| 121 | | | **For the following types of training (inservice/ continuing education), Do staffs from this facility attended;** | | | **Within the past 12 months?** | | | | | | | **Within the past five years?** | | | | | | G2 | |
|  |  |  |  |  |  | No (0) | | | | Yes (1) | | | No (0) | | | | Yes (1) | |  | |
| 121a | | | IUD insertion Skills | | |  | | | |  | | |  | | | |  | | G2a | |
| 121b | | | Family Planning counseling | | |  | | | |  | | |  | | | |  | | G2b | |
| **SERVICE PROVISION** | | | | | | | | | | | | | | | | | | |  | |
|  | | | **Do the following services have been provided at least once within the past one month at this facility?** | | | | | | | | No (0) | | | | | Yes (1) | | | H | |
| 122a | | | Antenatal care | | | | | | | |  | | | | |  | | | H1a | |
| 122b | | | Normal delivery care | | | | | | | |  | | | | |  | | | H1b | |
| 122c | | | Early postpartum check up (for mother) within 7 days | | | | | | | |  | | | | |  | | | H1c | |
| 122d | | | Postpartum check up (for newborn/infant) | | | | | | | |  | | | | |  | | | H1d | |
| 122e | | | Advice, support, and promotion of breastfeeding | | | | | | | |  | | | | |  | | | H1e | |
| 122f | | | Family planning services | | | | | | | |  | | | | |  | | | H1f | |
| 122g | | | Counseling on maternal self-care during postpartum period | | | | | | | |  | | | | |  | | | H1g | |
| 122h | | | Counseling on newborn care/breastfeeding | | | | | | | |  | | | | |  | | | H1h | |
| 122i | | | Counseling on family planning | | | | | | | |  | | | | |  | | | H1i | |
| 123 | | | **Are the following services provided every day that this facility is open?** | | | | | | | | No (0) | | | | | Yes (1) | | | H2 | |
| 123a | | | Antenatal care | | | | | | | |  | | | | |  | | | H2a | |
| 123b | | | Normal delivery care | | | | | | | |  | | | | |  | | | H2b | |
| 123c | | | Postpartum care services (for mother) | | | | | | | |  | | | | |  | | | H2c | |
| 123d | | | Family planning services | | | | | | | |  | | | | |  | | | H2d | |
| 123e | | | Post abortion care | | | | | | | |  | | | | |  | | | H2e | |
| 123f | | | PMTCT | | | | | | | |  | | | | |  | | | H2f | |
| **EMERGENCY SERVICES AND REFERRAL** | | | | | | | | | | | | | | | | | | |  | |
| **Select NO or YES answers for the following** | | | | | | | | | | | **No (0)** | | | | | **Yes (1)** | | |  | |
| 124a | | | **Is a skilled attendant for maternity services available on site, 24 hours/day, 7 days/week? By “Skilled attendant” I mean a physician, Health officer, midwife or nurse.** | | | | | | | |  | | | | |  | | | H3a | |
| 124b | | | **Is a skilled attendant for maternity services available on call 24 hours/day, 7 days/week?** | | | | | | | |  | | | | |  | | | H3b | |
| 124c | | | **Are services for caesarean section available 24 hours/day, 7 days/week?** | | | | | | | |  | | | | |  | | | H3c | |
| SUPPLIES | | | | | | | | | | | | | | | | | | | | |
| 125 | | | **How are the quantities of drugs and medical supplies needed by this facility determined?**  *Tick one best response.* | | | | | 1. Quantities determined at national level (e.g. standard drug kit) 2. Quantities determined at district level 3. Quantities determined through supervisory visits 4. Quantities determined by facility staff   Other (specify): _________ | | | | | | | | | | | | I1 |
| 126 | | | **Are maternity clients required to purchase/provide supplies/drugs at time of delivery?** | | | | | 1. No 2. Yes | | | | | | | | | | | | I2 |
| 127 | | | **Are FP clients required to purchase/provide the following supplies?** | | | | | No(0) | | | | | | | Yes(1) | | | | | I3 |
| 127a | | | Gloves | | | | |  | | | | | | |  | | | | | I3a |
| 127b | | | IUD | | | | |  | | | | | | |  | | | | | I3b |
| 127c | | | Soap | | | | |  | | | | | | |  | | | | | I3c |
| 127d | | | Medications/Medicine | | | | |  | | | | | | |  | | | | | I3d |
| 127e | | | Sanitary pads | | | | |  | | | | | | |  | | | | | I3e |
| 127f | | | Antiseptic liquid | | | | |  | | | | | | |  | | | | | I3f |
| 127g | | | Gauze/cotton | | | | |  | | | | | | |  | | | | | I3g |
| **CLINICAL MANAGEMENT GUIDELINES & PROTOCOLS** | | | | | | | | | | | | | | | | | | | |  |
|  | | | **Are the following types of FP service delivery guidelines/clinical management protocols; 1) are available at the facility? 2) Clearly outline steps in management of complication?** | | | | | | **Available** | | | | | **clearly outline steps of management** | | | | | | j |
|  |  |  |  |  |  |  |  |  | No(0) | | | Yes(1) | | No(0) | | | | Yes(1) | |  |
| 128a | | | Guidelines for FP counseling | | | | | |  | | |  | |  | | | |  | | J1a |
| 128b | | | Family planning guidelines for service providers | | | | | |  | | |  | |  | | | |  | | J1b |
| 128c | | | Flowcharts on IUD insertion procedures | | | | | |  | | |  | |  | | | |  | | J1c |
| 128d | | | **Are these guidelines/protocols available for reference by staff at all times?**  *If NO:* **Why not?** | | | | | | 1. No, they are locked for safe keeping 2. No, staff keep them in their homes 3. Yes   other (specify): _________  99 Do not know | | | | | | | | | | | J1d |
| **PART II OBSERVATION** | | | | | | | | | | | | | | | | | | | | code |
| **INFRASTRUCTURE AND EQUIPMENT** | | | | | | | | | | | | | | | | | | | |  |
| **Which of the following items are available and in satisfactory condition?**  *Be sure to look at each item. Code as unsatisfactory items which in your judgment are not functional, have missing parts, are unhygienic, or otherwise sub-standard.* | | | | | | | | | | | | | | | | | | | | **K** |
| 129a | | **Examination room or area providing client privacy (room for screening, counseling, and examination)** | | | 1. Not available 2. Available but not satisfactory 3. Available and satisfactory | | | | | | | | | | | | | | | K1a |
| 129b | | **Examination table** | | | 1. Not available 2. Available but not satisfactory 3. Available and satisfactory | | | | | | | | | | | | | | | K1b |
| 129c | | **Storage area or cupboard for drugs and other supplies** | | | 1. Not available 2. Available but not satisfactory 3. Available and satisfactory | | | | | | | | | | | | | | | K1c |
| 129d | | **Toilet facilities or latrine** | | | 1. Not available 2. Available but not satisfactory 3. Available and satisfactory | | | | | | | | | | | | | | | K1d |
| 129e | | **FP service room** | | | 1. Not available 2. Available but not satisfactory 3. Available and satisfactory | | | | | | | | | | | | | | | K1e |
| 129f | | **Delivery bed/couch** | | | 1. Not available 2. Available but not satisfactory 3. Available and satisfactory | | | | | | | | | | | | | | | K1f |
| 129g | | **Water supply** | | | 1. Not available 2. Available but not satisfactory 3. Available and satisfactory | | | | | | | | | | | | | | | K1g |
| 129h | | **Which of the following does the labor/FP room usually have for light supply?** | | | - 1. None   2. Electricity   3. Candles   4. Kerosene lamp   5. Battery torch with working batteries available   6. Generator   7. Solar power   Other (specify): _________  99 No labor room | | | | | | | | | | | | | | | K1h |
| **BASIC EQUIPMENTS** | | | | | | | | | | | | | | | | | | | |  |
| **Which of the following items are available and in satisfactory condition? L**  *Be sure to* ***look*** *at each item.* | | | | | | | | | | | | | | | | | | | | |
| 130a | | **Speculum (various sizes)** | | 1. Not available 2. Available but not satisfactory 3. Available and satisfactory | | | | | | | | | | | | | | | | L1a |
| 130b | | **Containers with lids to store boiled instruments, gloves, etc.** | | 1. Not available 2. Available but not satisfactory 3. Available and satisfactory | | | | | | | | | | | | | | | | L1b |
| 130c | | **Containers with lids for disposal of sharps** | | 1. Not available 2. Available but not satisfactory 3. Available and satisfactory | | | | | | | | | | | | | | | | L1c |
| 130d | | **Foley catheter & urine collection bags** | | 1. Not available 2. Available but not satisfactory 3. Available and satisfactory | | | | | | | | | | | | | | | | L1d |
| 130e | | **Stretchers** | | 1. Not available 2. Available but not satisfactory 3. Available and satisfactory | | | | | | | | | | | | | | | | L1e |
| 130f | | **Lockable storage area or cupboard for FP equipment** | | 1. Not available 2. Available but not satisfactory 3. Available and satisfactory | | | | | | | | | | | | | | | | L1f |
| 130g | | **Kelly clamp( special IUCD inserter clamp)** | | 0 Not available  1 Available but not satisfactory  2 Available and satisfactory | | | | | | | | | | | | | | | | L1g |
| 130h | | **Elbow size glove** | | 0 Available but not satisfactory  1 Available and satisfactory  2 Not available | | | | | | | | | | | | | | | | L1h |
| 130i | | **Sponge forceps** | | 1. Not available 2. Available but not satisfactory 3. Available and satisfactory | | | | | | | | | | | | | | | | L1i |
|  | | **Educational materials** | | | | | | | | | | | | | | | | | | M |
| 131a | | **What types of client information, education and communication**  **(IEC) materials are available today for FP service?** | | Specify:  1.  2.  3. | | | | | | | | | | | | | | | | M1 |
| **Which of the following IEC and/or counseling materials are available?** *Be sure to* ***look*** *at each material to determine whether or not the following topics are covered.* | | | | | | | | | | | | | | | | | | | | **M2** |
| 132a | | **On postpartum care (self-care following delivery)** | | 1. Not seen 2. Seen at this facility | | | | | | | | | | | | | | | | M2a |
| 132b | | **On family planning** | | 1. Not seen 2. Seen at this facility | | | | | | | | | | | | | | | | M2b |
| 132c | | **On postabortion care (self-care, postabortion, family planning)** | | 1. Not seen 2. Seen at this facility | | | | | | | | | | | | | | | | M2c |
| 132d | | **On sexually transmitted infections/HIV/AIDS** | | 1. Not seen 2. Seen at this facility | | | | | | | | | | | | | | | | M2d |
| 132e | | **On LACMs (long acting contraceptive methods )** | | 1. Not seen 2. Seen at this facility | | | | | | | | | | | | | | | | M2e |
| 132f | | **On other FP topics (specify)** | | 1. Not seen 2. Seen at this facility | | | | | | | | | | | | | | | | M2f |
| **ESSENTIAL DRUGS AND CONSUMABLE SUPPLIES** | | | | | | | | | | | | | | | | | | | | **M3** |
| *For essential drugs and consumable supplies, be sure to* ***look*** *at each item, to see if it is available, is in good condition and has not expired.* | | | | | | | | | | | | | | | | | | | |  |
| **Consumable supplies** | | | | | | | | | | | | | | | | | | | | |
| 133a | | **Gloves** | | 1. Not available 2. Available but not satisfactory 3. Available and satisfactory | | | | | | | | | | | | | | | | M3a |
| 133b | | **Disposable syringes and needles** | | 1. Not available 2. Available but not satisfactory 3. Available and satisfactory | | | | | | | | | | | | | | | | M3b |
| 133c | | **Oxygen** | | 1. Not available 2. Available but not satisfactory 3. Available and satisfactory | | | | | | | | | | | | | | | | M3c |
| 133d | | **IV kit** | | 1. Not available 2. Available but not satisfactory 3. Available and satisfactory | | | | | | | | | | | | | | | | M3d |
| 133e | | **IV catheter sets** | | 1. Not available 2. Available but not satisfactory 3. Available and satisfactory | | | | | | | | | | | | | | | | M3e |
| **Drugs (Contraceptives)** | | | | | | | | | | | | | | | | | | | | **M4** |
| 134a | | **Oral contraceptives (any type)** | | 1. Not available 2. Available but not satisfactory 3. Available and satisfactory | | | | | | | | | | | | | | | | M4a |
| 134b | | **Injectable contraceptives (any type)** | | 1. Not available 2. Available but not satisfactory 3. Available and satisfactory | | | | | | | | | | | | | | | | M4b |
| 134c | | **Condoms** | | 1. Not available 2. Available but not satisfactory 3. Available and satisfactory | | | | | | | | | | | | | | | | M4c |
| 134d | | **IUCDS/IUDS** | | 1. Not available 2. Available but not satisfactory 3. Available and satisfactory | | | | | | | | | | | | | | | | M4d |
| 134e | | **Implants** | | 1. Not available 2. Available but not satisfactory 3. Available and satisfactory | | | | | | | | | | | | | | | | M4e |
| 134f | | **Emergency contraceptive pills** | | 1. Not available 2. Available but not satisfactory 3. Available and satisfactory | | | | | | | | | | | | | | | | M4f |
| **Drugs (Intravenous solutions)** | | | | | | | | | | | | | | | | | | | | **M5** |
| 135a | | **Sterile water for injection** | | 1. Not available 2. Available but not satisfactory 3. Available and satisfactory | | | | | | | | | | | | | | | | M5a |
| 135b | | **Sodium chloride/saline solution** | | 1. Not available 2. Available but not satisfactory 3. Available and satisfactory | | | | | | | | | | | | | | | | M5b |
| 135c | | **Sodium lactate compound solution (Ringer’s lactate)** | | 1. Not available 2. Available but not satisfactory 3. Available and satisfactory | | | | | | | | | | | | | | | | M5c |
| 135d | | **Glucose with sodium chloride** | | 1. Not available 2. Available but not satisfactory 3. Available and satisfactory | | | | | | | | | | | | | | | | M5d |
| **Disinfectants, antiseptics & cleansers** | | | | | | | | | | | | | | | | | | | | **M6** |
| 136a | | **Alcohol** | | 1. Not available 2. Available but not satisfactory 3. Available and satisfactory | | | | | | | | | | | | | | | | M6a |
| 136b | | **Iodine** | | 1. Not available 2. Available but not satisfactory 3. Available and satisfactory | | | | | | | | | | | | | | | | M6b |
| 136c | | **Chlorhexidine** | | 1. Not available 2. Available but not satisfactory 3. Available and satisfactory | | | | | | | | | | | | | | | | M6c |
| 136d | | **Soap** | | 1. Not available 2. Available but not satisfactory 3. Available and satisfactory | | | | | | | | | | | | | | | | M6d |
| 136e | | **0.5 chlorine solution (bleach/JIK)** | | 1. Not available 2. Available but not satisfactory 3. Available and satisfactory | | | | | | | | | | | | | | | | M6e |
| 136f | | **Formaldahyde (8%) or Glutaraldehyde (2%)** | | 1. Not available 2. Available but not satisfactory 3. Available and satisfactory | | | | | | | | | | | | | | | | M6f |

**Thank you very much for your valuable information!**
